# Acute Suffocative Œdema of the Lungs

**Published:** 1927

**Authors:** J. Anthony Birrell

**Affiliations:** Physician, Children's Hospital, and Assistant Physician, General Hospital, Bristol


### ACUTE SUFFOCATIVE ŒDEMA OF THE LUNGS.

BY

J. Anthony Birrell, M.D., M.R.C.P. Lond.,
*Physician, Children's Hospital, and Assistant Physician, General Hospital, Bristol.*

---

Welch's[1] theory of the cause of this condition, which according to Osler is that generally accepted, is to the effect that the quantities of blood dealt with by the right and left hearts in any given unit of time must be equal; if not, then there must be a turgescence of either the pulmonary or the systemic circulation. In his words, in acute suffocative œdema "there is a disproportion between the work done by the left heart and that done by the right." If the amount of blood drawn from the lungs through the pulmonary veins is less than that delivered to them through the pulmonary artery, then the condition of affairs in the pulmonary circulation is such as will cause this dangerous emergency. Welch's experimental work on rabbits showed that after compression of the pulmonary veins or massage of the left ventricle so as to produce fibrillation, acute pulmonary œdema sometimes ensued. He was unable to induce the condition by simple compression of the aorta, and therefore argued that, unless there was some pathological change in the left ventricle such as might be brought about by massage

[1] Welch, *American Medicine*, 1904, viii., 195.

of the exposed organ, or direct interference with the pulmonary circulation due to obstruction of the pulmonary veins, the balance between the right and left hearts was maintained, and that direct interference with the left heart might affect that balance.

In the dyspnœic crises under discussion the patient is middle-aged or elderly, and there has been perhaps nothing to cast suspicion on the integrity of the circulation hitherto. Suddenly, and, in those cases which I have seen, towards the end of the day, these patients are taken with extreme dyspnœa, feeling that they are being suffocated by an obstruction in the air passages. On my arrival I have found them always sitting up in bed or in a chair, breathing in an anxious, laboured way, incapable of speech as a result of the dyspnœa and of the embarrassment caused by efforts to spit out a pink frothy mucus welling up from the trachea : the attempts to cough this up are feeble and futile, or non-existent. The gaze is terrified and staring, being with difficulty turned towards one ; the face and general body surface white or grey, and cold with a clammy sweat : there is no cyanosis or venous distension. Here is a condition differing from a sudden attack of asthma in which there is no sputum, and from right heart failure where we are accustomed to see cyanosis and engorgement of the veins of the neck and upper arms. The pulse is usually 100–120, of very small, almost imperceptible, volume. The heart sounds are rendered inaudible by râles throughout the lungs, most numerous towards the bases ; I have not been able to make out any increase in the area of right heart dullness.

When one is confronted with an emergency of this kind, engorgement of the systemic veins, which might be taken as an indication for venesection, a measure recommended by many authorities for this condition, is seen not to be present. Judged on that basis alone, therefore, this operation would seem to be futile ; but a moment's reflection will show that this deduction is incorrect. The veins are not full because, whilst the right heart is passing on the blood from them into the lungs, they are not being fed through the capillaries from the arterial side of the systemic circulation, since the left heart is not delivering enough blood through to them. There is, as it were, a traffic block in the lung capillaries. More blood has entered the lungs by the pulmonary artery than can get through, and a " jam " results, so that the quantities of blood emerging through the left heart and aorta are diminished below the normal ; for some reason the balance between the right and left hearts is upset. In fact, to carry the analogy farther, the traffic control has broken down. How it is that this control of balance of output between the two sides of the heart is maintained is a matter of speculation, but perhaps it may be an affair of nervous synchronisation ; for many cases of left heart failure of considerable duration are seen where acute pulmonary œdema does not take place (unless perhaps immediately before death), and I would suggest that its absence depends on some nervous inhibitory action controlling the right heart, so that it does not pump too much blood through into the lungs. In the cases I shall quote it seems to be clear that there were considerable degrees of left heart inadequacy without acute pulmonary œdema immediately ensuing. In the same cases, moreover, after repeated attacks of pulmonary œdema, this left heart insufficiency seemed to be even more marked than before, and yet there were no subsequent attacks, so that the right heart must surely have been under the influence of some inhibitory control. It is the sudden cessation of this inhibitory control (if such there be), together with a weakening left heart, which seems to determine the onset of this condition.

We are therefore faced with the task of attempting to relieve the blood block in the lung capillaries and of controlling the amount of work done by the right heart. Nature makes an effort in the former direction, as is shown by the exudation of fluid through the bronchial tree and its discharge through the trachea, but the respiratory exchange is thereby interfered with, and the patient is in danger of suffocation. By venesection some of the blood entering the jammed lung capillaries is diverted, that is to say, some control is brought to bear on the work being done by the right heart, not very much perhaps, but enough to start the relief of the block. Moreover, this relief is being acquired to some further extent by rendering the exit from the block the more easy through the left heart; for it will be seen that as this block becomes less congested there is evidence of more blood coming through, in that there is an increase of the bleeding from the *distal* end of the incised vein. With the circulation picking up, this gathering stream of blood attains greater dimensions as the left heart passes on the blood from the previously jammed lung capillaries: the traffic control is given an opportunity to reassert itself, and the circulation proceeds in an orderly fashion as before.

In support of this reasoning I have made the following observations on some cases in which I have performed venesection. On looking for a vein in the antecubital fossa it is difficult to locate one, but after the upper arm has been constricted and the front of the forearm massaged upwards, the median basilic can usually be identified at the bend of the elbow. On its exposure by incision of the skin and isolation for the distance of about an inch from the surrounding structures it appears to be almost empty. After ligatures have been slipped under the upper and lower extremities of the isolated segment the vessel is incised in a longitudinal direction between these. At first blood flows but slowly in dark drops ; one must expect here no sudden gush of blood as in a case of right heart failure. In a little while this drip quickens into a trickle which soon becomes a stream of red blood. In each case I have noted that the discharge comes from the distal end of the vein ; compression of the vessel in the forearm will cause the flow to cease, whereas compression in the upper arm proximal to the incision has no effect.

I have imagined that this acceleration in the venesection flow results from the circulation picking up through the arterial tree and coming on through the capillaries to the vein. The gradual return of the natural pinkness to the previously ashen features, and a flow of healthy colour to the cheeks, also coincides with this return of the capillary circulation to normal. With these changes there comes on a cough which is to be hailed as a good omen ; the breathing is freer and the patient gasps his relief.

The amounts of blood I have drawn off have varied from fourteen to twenty-four ounces. I have allowed bleeding to continue as long as the condition is improving and the pulse gaining in volume and slowing. If it quickens or appears to be losing some of its newly-acquired strength, the bleeding is stopped. By now the patient should be of good colour, able to hold his breath and cough, and lie back against his pillows breathing with tranquillity.

In less urgent cases, in place of venesection I have injected morphine and atropine hypodermically, and relief has been quickly brought about. I suggest that it is the vaso-depressor action of these drugs which is the important factor in their utility here, but I have not taken a series of blood pressure observations in these cases. Chloroform inhalations likewise have been quoted as a useful remedy.

The following are notes of two cases on whom venesection was practised:—

**Case 1.**—Mrs. H., aged 71. In the evening of May 30th, 1926, I was called to see this woman, and found her suffering from a typical acute suffocative attack such as I have described. She was cold, grey and almost pulseless, with numerous râles throughout both lungs, and on opening the vein a few drops only of blood was I able to express by massaging the forearm upwards. The flow soon increased, however, from the distal end of the vein, and I ultimately relieved her of a total volume of eighteen ounces. When this had been done she was very much better, and able to lie back in bed and tell me that she had recently survived three similar, but much less severe, attacks, each associated with a blood-stained, frothy sputum.

Next day when I saw her the pulse was regular and strong, 90 in rate, the blood pressure 190, the apex beat 4½ inches from the mid-line, the cardiac sounds weak and tick-tack, but the lungs quite clear.

**Case 2.**—Mr. S., aged 63, tram cleaner. I attended this man in 1925, when he suffered from auricular fibrillation, the apex beat being in the sixth space and 5 inches from the mid-line. There were no murmurs, and the feet were not œdematous. In 1926, although the fibrillation had ceased under digitalis, he became dyspnœic on any exertion and a systolic murmur was audible at the apex, which I attributed to ventricular dilatation. One evening towards the end of March, 1927, he had an attack of acute suffocative œdema of the lungs. The pulse was 125, regular, and the blood pressure was 210/135. He seemed to be *in extremis*, but, nevertheless, I withdrew forthwith twenty ounces of blood and he at once improved, the pressure falling in these few minutes to 190/110, and the pulse slowing to 107.

He got about, against my advice, during the next few days, and with the subsequent rise in blood pressure to 200/125 another attack, which I thought was going to prove fatal, supervened at 10 p.m., five days after the first one. In addition to the other signs there were now many extra-systoles. I drew off twenty-four ounces of blood, thereby reducing the pressure to 160/105, and he was much improved, so that next morning the breathing was normal and the lungs free of râles. Unfortunately sepsis set in at the site of venesection, and I had him removed to hospital, where he died, I believe, in a subsequent attack.

In the next two cases, although there were undoubtedly signs of impending attacks of suffocative œdema from time to time, whenever I saw them the conditions did not seem urgent enough to warrant venesection, and I employed other measures to bring about relief.

**Case 3.**—Mr. P., aged 51, draughtsman. In May, 1925, this man had a sudden paresis of the left side of the body which gradually passed off: there had been a similar transient hemiparesis four years previously.

In August, 1925, he was taken with a sudden dyspnœa just after going to bed; I found him sitting up pallid and sweating, very dyspnœic, with a feeble quick pulse and the chest full of râles, the bronchial exudate rattling in the trachea. I injected gr. 1/100 of atropine and found him next morning to be much better, with a pulse of 84 and the chest clear of râles. The blood pressure was 185/110, there was some albumin in the urine and a marked albuminuric retinitis.

During the course of the next few weeks, just after going to bed, there were several attacks of shortness of breath and "whistlings in the chest," as his wife described them, but neither their severity nor their duration caused her to send for treatment.

Later in the year, against my advice, he tried to resume work, but found that the effort of walking to his office caused such dyspnœa that he discontinued his attempt. I found no râles in his chest one morning when he walked to see me, neither was there any œdema of the feet, and yet there was an extreme dyspnœa, with a pulse of 110 lacking any evidence of respiratory rhythm. This shortness of breath, he said, had come on just before arriving at my house, and he thought it was due to walking a little too quickly. He was not a fat man, and the apex beat could be localised about 4 inches from the mid-line. He now became confined to his house, and hemorrhages appeared in his retinæ.

In the early part of 1926 the attacks of threatened pulmonary œdema became more frequent and severe, so that I was often called to see him towards midnight. The lungs would then be full of râles, a little blood-stained sputum welling from his mouth, but relief always seemed to follow an injection of atropine, so that venesection was not needed, and next morning the breathing would be peaceful and the lungs free of adventitious sounds. He then developed a dry pericardial friction rub over the left ventricle and had precordial pain in these dyspnœic attacks, and a month later died sitting up in bed and gasping for breath before I could get to him, without ever having had any cyanosis or œdema of his legs, as far as I know.

**Case 4.**—Mrs. G., aged 49. This woman had a hemiparesis in November, 1926, from which she gradually recovered. I subsequently noted that the pulse was alternating, 80 in rate, exhibiting respiratory rhythm, with a blood pressure of 210/150. The discs were œdematous, and there was some albumin in the urine. The Wassermann reaction was negative.

In April, 1927, the blood pressure had risen to 230/160, and there was what I took to be a threatened attack of pulmonary œdema, with râles in the lungs and the typical bronchial exudate, one evening after she had gone to bed. I injected gr. 1/150 of atropine with gr. 1/4 of morphine, and these symptoms subsided in a couple of hours. I noted a few days later that the pulse had become quick (120) and perfectly regular, having lost its respiratory variations; the blood pressure was the same as before.

In May there were two typical attacks of acute pulmonary œdema, which again subsided after atropine and morphine.

In June, when I was seeing her frequently, the blood pressure fell in the course of a few days to 170/140, the heart meanwhile dilating so that the apex beat, which had previously been but little out from the normal, went out to about 6 inches from the mid-line in the sixth space, the pulse being 120 and the heart sounds of the gallop type. A fortnight later I noted that the blood pressure had fallen to 150/115, and a few days afterwards death ensued without another attack of pulmonary œdema, and unaccompanied by cyanosis or œdema of the extremities.

### SUMMARY.

1. In acute suffocative pulmonary œdema the blood flows centripetally from the distal end of the incision in the vein, which is the reverse of that which occurs in venesection for right heart failure.

2. There is no systemic œdema in these cases.

3. In all four cases investigated there was high blood pressure associated with ventricular inadequacy. In support of this contention may be noted the weakness of the heart sounds in Case 1 and the mitral systolic murmur in Case 2, presumably due to a relative incompetence consequent on stretching of the auriculo-ventricular ring from weakening of the ventricular muscle as a whole. In Case 3, likewise, the marked dyspnœa with tachycardia, occurring without any cyanosis or râles in the lungs, was apparently due to left ventricle failure, since nothing else could be found to account for it. In Case 4 clear evidence of left ventricle defeat seems to be established by the dilatation of the ventricle and the accompanying fall in blood pressure, together with narrowing of the pulse pressure.

4. That something more than left ventricular failure alone is necessary to determine the actual onset of acute pulmonary œdema seems to be evident from a study of Cases 2 and 4, for in the former case a relative mitral incompetence was present for some months before an attack of pulmonary œdema ensued, and in the latter case, when the ventricle gave way and the blood pressure was falling, there were no subsequent attacks.

5. The immediate object in treatment is to relieve the distension of the lung capillaries quickly, by diminishing the blood pressure, either mechanically by bleeding or in less urgent cases by vaso-depressor drugs, such as morphine, atropine or chloroform. By these measures the left heart also is automatically relieved of some of its load.

6. As the blood pressure reacts to these manœuvres and becomes lower and the left heart is thereby relieved, it is capable of recovering, and of maintaining that recovery for some time, drawing through from the lungs the surplus blood which is distending the lung capillaries and threatening the patient's life, and so furnishing an opportunity for the balance of output of both sides of the heart to be restored.

